# Assessment of students’ perspective on introduction of “digital dentistry” as self- directed learning module in undergraduate prosthodontics curriculum: A mixed-method study

**DOI:** 10.1016/j.jobcr.2025.06.010

**Published:** 2025-06-22

**Authors:** Shuchi Tripathi, R.K. Dixit, Suyog Sindhu, R.D. Singh, Rameshwari Singhal, Richa Khanna

**Affiliations:** a Department of Prosthodontics, King George's Medical University, UP, Lucknow, India; b Department of Pharmacology, King George's Medical University, UP, Lucknow, India; c Department of Periodontology, King George's Medical University, UP, Lucknow, India; d Department of Pedodontics and Preventive Dentistry, King George's Medical University, UP, Lucknow, India

**Keywords:** Curriculum, Digital dentistry, Perspective, Module, Self- directed learning

## Abstract

**Introduction:**

Innovative approaches to curriculum design and implementation are critical in meeting the needs of today's students. The present study was undertaken to determine the perspective of students on the introduced module of ‘Digital Dentistry’ along with knowing their self –directed learning abilities.

**Methods:**

The present study was conducted in the Medical University after ethical approval was obtained. Forty volunteers from 3rd and 4th year undergraduates of “Bachelor of Dental Surgery” course were selected and consent form was obtained. A pre-validated questionnaire on self-directed learning (SDLI) was administered to the volunteers to know their SDL abilities. A pre-post-session questionnaire containing feedback questionnaires was prepared, validated and administered before the session. A pre designed module was applied on the volunteers by using SDL session. 2 h duration of First contact session followed by 2 weeks of intersession period, where student went through the shared online/offline study material and visit to the lab under faculty supervision. Outcome assessment was done with 30 MCQ based on the 3 chapters taught in the session. In Second contact session, debriefing by faculty was done and post-module questionnaire was administered. The responses obtained from SDLI, assessment questionnaires and pre-post-module questionnaires were further analyzed statistically.

**Results:**

Student's SDLI score with median value of 77.325 represented a good score, with learning motivation scored highest in SDLI. The total score significantly improved post-intervention, with the mean score increasing from 28.83 (5.43) pre-intervention to 35.35 (4.36) post-intervention.

**Conclusion:**

Significant improvement in knowledge, awareness and perceptions of digital dentistry was seen after the conduct of the session.

## Graphical abstract

**Figure:**
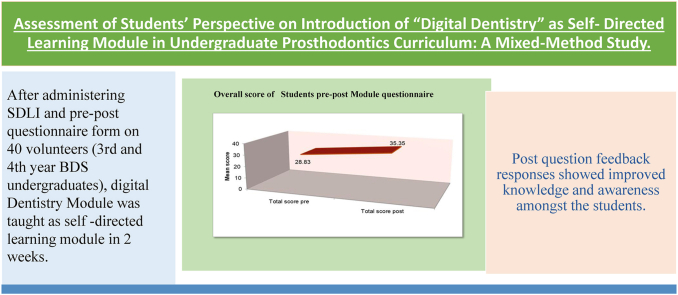
Graphical presentation of the article

## 1 Introduction

Digitalization in dentistry is routinely used to improve the accessibility and exchange of documents, facilitate the collaboration and communication among various stakeholders,^1^ improving clinical efficiency and improving the overall outcome, whether in education or patient treatment. Beyond the clinical applications, digital simulation systems are beneficial for education and training.^2^^,^^3^ The students of today are considered “digital natives”; therefore, working with digital tools is easy and enjoyable for them.^4^ Digitization offers increased accuracy, efficiency, and cost-effectiveness compared to manual methods.^5^ In India, education of dental students is still solely focused on conventional teaching methods including traditional laboratory techniques such as waxing, casting, finishing, and tooth preparation exercises on the phantom head. A dental institution that relies heavily on teaching traditional restorative procedures may particularly face many challenges to embrace new technology.^6^

Self-directed learning (SDL) as defined by Knowles is a process in which individuals take the initiative with or without the help of others in diagnosing their learning needs, setting their own learning goals, identifying appropriate learning resources, and selecting appropriate learning strategies.^7^ Self-directed learning as a defined teaching pedagogy is commonly used since 1960 and can be used in classroom and experiential settings. SDL adds variety to teaching-learning methods and provides an option for curriculum makers to choose this method in alignment with some learning objectives. The conduct of SDL is quite variable at different places.^8^^.^^9^ Self-directed learning can potentially save time in an existing curriculum schedule by allowing individuals to learn at their own pace, focusing on areas of interest, and utilizing diverse learning resources which is not possible in traditional method. When teaching individuals who are new to this model, care must be taken to appropriately scaffold and structure learning to develop the underlying soft skills needed for students to be successful as self-directed learners. During its implementation in a classroom setting, challenges are faced both by the learner and the educator. Thus, Faculty members should proactively plan for potential challenges during the course design process.

An instructional Module of ‘Digital Dentistry’ was developed to sensitize the undergraduate students with digital dentistry after need assessment of target group .^10^ The module was flexible enough to be adopted by dental institutions depending upon infrastructure, manpower and support.^10^ As self-directed learning will help the students to learn at their own pace and require less time of classroom teaching as compared to traditional teaching, the present project on ‘Assessment of Students’ Perspective on Introduction of “Digital Dentistry” as Self- Directed Learning Module in Undergraduate Prosthodontics Curriculum: A Mixed-Method Study.’ was undertaken to determine the perspective of students on the introduced module along with understanding the self – directed learning abilities of the students.

## 2 Methods

The present study was conducted in the Prosthodontics department of the institution after IEC approval (approval no- 3506/ethics/2024). 40 volunteers (provided consent to be included in the study via google form) from third year and fourth year of undergraduates of “Bachelor of Dental Surgery (BDS)” course were selected. A core team of 3 faculty members from department of Prosthodontics, was formed to be directly involved in conducting the study starting from teaching the students in lecture theatre, facilitating their laboratory visits and recording their assessment.

A pre-validated questionnaire on self-directed learning (SDLI) was administered to the volunteers to know their SDL abilities.^11^ The questionnaire consisted of 20 items, of which the first 6 items were related to exploring students’ learning motivation, 7–16 exploring planning and implementation abilities, and the remaining 4 dealt with their interpersonal communication skills. The participants were asked to select from a 5-point Likert scale: “strongly disagree,” “disagree,” “neutral,” “agree,” and “strongly agree.”

A pre-post-session questionnaire containing feedback questionnaires, based on 5-point-Likert scale, to know the students' knowledge and view on “Digital Dentistry” was developed and content was face validated with the help of 6 faculty members (2 faculty from Prosthodontics department, 1 from orthodontics department, one from Pharmacology department, one from Microbiology department and one faculty member from Geriatric Mental Health department). They all were actively involved in medical education with more than 10 years of academic experience. A google form was prepared after validation, in which 8 questions were based on 5-point Likert's scale and 2 questions were framed as multiple option to know the knowledge of participants regarding the use of digital dentistry and barriers of digital dentistry. The pre-session questionnaire also contained an open-ended question related to students' expectations from the module and post-session questionnaire included an open-ended question related to their feedback of the implemented module. The reliability analysis of the questionnaire scale was measured by pilot testing of the questionnaire on 15 students and Cronbach's Alpha, which indicated good internal consistency with a value of 0.804. The standardized items also showed a similar reliability score of 0.809 across 8 items, suggesting that the scale is reliable for assessing the intended construct.

After administering pre-module questionnaire, a pre designed module^10^ was applied on 40 volunteers from 3rd year and final year dental undergraduates. 2 h duration of lecture for ‘Digital Dentistry’∗(First contact session) was delivered by 2 faculty members. The lecture contained 3 chapters of the module on Introduction, diagnosis and treatment planning and Computer-Aided Design- Computer-Aided Manufacturing (CAD- CAM). The purpose of the lecture was to briefly orient the student regarding impact of digitalization in dentistry in various aspects. In further 2 weeks (intersession period), online/offline supplementary materials were shared with the students via WhatsApp to facilitate learning at their own pace.

**Fig. 1:**
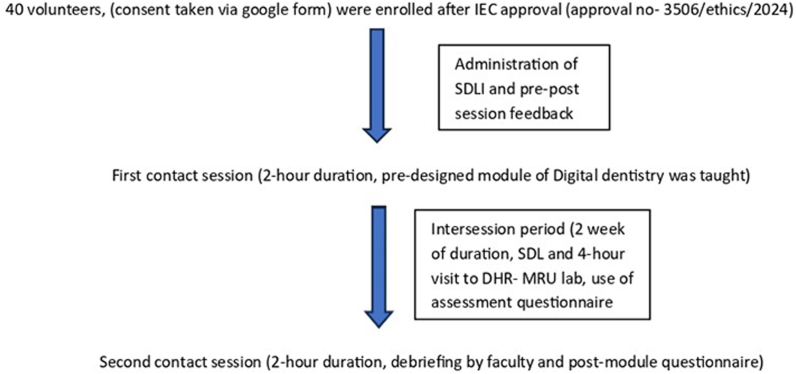
Conduct of SDL session.

During intersession period, 4-h visit to Department of Health research—Multidisciplinary Unit (DHR- MRU) lab was facilitated to the students under faculty supervision. In DHR lab, a demonstration of intraoral scanner (3 shape Trios scanner), heptic device (3D systems) and 3-D printing (Form 2) of maxilla was done. For outcome assessment, 3 end of module assessment questionnaires containing 10 multiple choice questions in each set, related to the chapters were prepared and were further face-validated by 6 Prosthodontics faculty members (subject experts) of the department. These assessment questionnaires were administered to the students in google form to evaluate their learning outcomes. 50 % passing score was decided to successfully complete the session. In Second contact session, debriefing by faculty was done and post-module questionnaire was administered. Fig. 1 summarizes the process of SDL session involved in the study.

The responses obtained from SDLI, assessment questionnaires and pre-post-module questionnaires were further analyzed statistically. Categorical variables were presented in number and percentage (%) and continuous variables were presented as mean and standard deviation (SD). Normality of data were tested by Kolmogorov-Smirnov test. If the normality is rejected then non parametric test were used. Quantitative variables were compared using Mann Whitney *U* test between two independent groups. Pre and post session responses were compared using Wilcoxon Signed Rank test. A p value of <0.05 was considered statistically significant. The data was entered in MS EXCEL spreadsheet and analysis was done using Statistical Package for Social Sciences (SPSS) version 23.0.

## 3 Result

Student's self-directed learning abilities was evaluated by using SDLI. Table 1 shows the result of self-directed learning scale amongst the students. It shows student's learning motivation scored highest amongst other 4 parameters. An analysis of the SDLI domains revealed that the participants showed strong learning motivation, with a mean score of 4.19. However, their planning and implementation skills were lower, with a mean score of 3.70. The participants demonstrated the ability to self-monitor their learning, with a mean score of 3.84 and their interpersonal communication skills were rated with a mean score of 3.71.

**Table 1:** Students response to Self-directed Learning Instrument (SDLI Median score −77.32).

S No.	Items	%disagreement	%Neutral	%agreement	Mean Score
**Learning Motivation**					
*1*	I know what I need to learn.	7.5	25	67.5	3.85
*2*	Regardless of the result or effectiveness of my learning, I still like learning.	5	12.5	82.5	4.1
*3*	I strongly hope to constantly improve and excel in my learning.	5	5	90	4.5
*4*	My successes and failures inspire me to continue learning.	5	7.5	87.5	4.28
*5*	I enjoy finding answers to questions.	5	7.5	87.5	4.3
*6*	I will not give up learning because I face some difficulties.	7.5	10	82.5.	4.08
	***Overall***	***5.83***	***11.25***	***82.9***	***4.19***
**Planning and Implementation**					
*7*	I can proactively establish my learning goals.	7.5	22.5	70	3.83
*8*	I know what learning strategies are appropriate for me in reaching my learning goals.	15	30	55	3.63
*9*	I set the priorities of my learning.	7.5	17.5	75	4.1
*10*	In the classroom or on my own, I am able to follow my own plan of learning.	12.5	40	47.5	3.55
*11*	I am good at arranging and controlling my learning time.	17.5	37.5	45	3.35
*12*	I know how to find resources for my learning.	17.5	25	57.5	3.58
*13*	I can connect new knowledge with my own personal experiences.	10	25	65	3.83
	***Overall***	***12.5***	***28.21***	***59.29***	***3.70***
**Self-Monitoring**					
*14*	I understand the strengths and weakness of my learning.	2.5	27.5	70	4
*15*	I can monitor my learning progress.	5.1	17.9	76.9	3.83
*16*	I can evaluate on my own learning outcomes.	2.5	45	52.5	3.68
	***Overall***	***3.37***	***30.13***	***66.5***	***3.84***
**Interpersonal communication**					
*17*	My interaction with others helps me plan for further learning.	12.5	10	77.5	3.83
*18*	I would like to learn the language and culture of those whom I frequently interact with.	2.5	22.5	75	3.9
*19*	I am able to express messages effectively in oral presentations.	15.4	46.2	38.4	3.29
*20*	I am able to communicate messages effectively in writing.	5	30	65	3.8
	***Overall***	***8.85***	***27.18***	***63.98***	***3.71***

**Graph 1:**
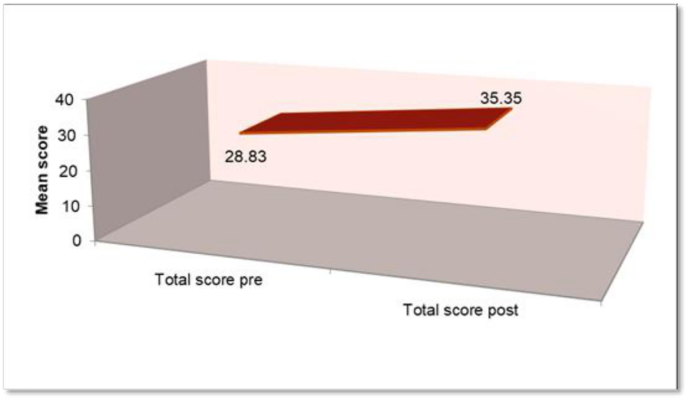
Overall score of Students pre-post Module questionnaire.

In Table 2, Participants showed significant improvement in their awareness and perceptions of digital dentistry. In Graph 1, the total score significantly improved post-intervention, with the mean score increasing from 28.83 (5.43) pre-intervention to 35.35 (4.36) post-intervention. The mean difference was −6.68, reflecting a 23.16 % increase (95 % CI: −8.54 to −4.51, Z = −4.92, p < 0.001).

**Table 2:** Students pre-post Module questionnaire responses.

	Mean	Total students (N)	SD	Mean difference	% Mean change	95 % CI of the difference	Z value	p-value
**I am aware with the term ‘Digital Dentistry”**								
Pre	3.325	40	1.14102	−1.15	−34.59	−1.49 to −0.81	−4.74	**<0.001**
Post	4.475	40	0.67889					
**I understand that Digital Dentistry is gaining popularity nowadays**								
Pre -	3.825	40	1.00989	−0.775	−20.26	−1.09 to −0.46	−4.05	**<0.001**
Post	4.6	40	0.67178					
**I agree that digital dental technologies can bring clarity in communication-**								
Pre	3.825	40	0.81296	−0.475	−12.42	−0.77 to −0.18	−2.92	**0.003**
Post	4.3	40	0.79097					
**According To me, my knowledge with respect to “digital dentistry”**								
Pre	2.525	40	0.96044	−1.425	−56.44	−1.76 to −1.09	−5.02	**<0.001**
Post	3.95	40	0.78283					
**According to me, “Digital Dentistry” is required in preclinical courses of Prosthodontics**								
Pre	3.675	40	1.24833	−0.8	−21.77	−1.24 to −0.36	−3.2	**0.001**
Post	4.475	40	0.78406					
**According to me, “Digital Dentistry” is required in clinical courses of Prosthodontics**								
Pre	4.125	40	1.06669	−0.5	−12.12	−0.89 to −0.11	−2.42	**0.016**
Post	4.625	40	0.66747					
**I am aware with Computer-aided design and computer-aided manufacturing (CAD/CAM) technology-**								
Pre	3.325	40	1.18511	−0.975	−29.32	−1.41 to −0.54	−3.65	**<0.001**
Post	4.3	40	0.82275					
**I am willing to adopt “digital dentistry” in my future clinical practice –**								
Pre	4.2	40	0.85335	−0.425	−10.12	−0.75 to −0.1	−2.44	**0.015**
Post	4.625	40	0.70484

**Graph 2:**
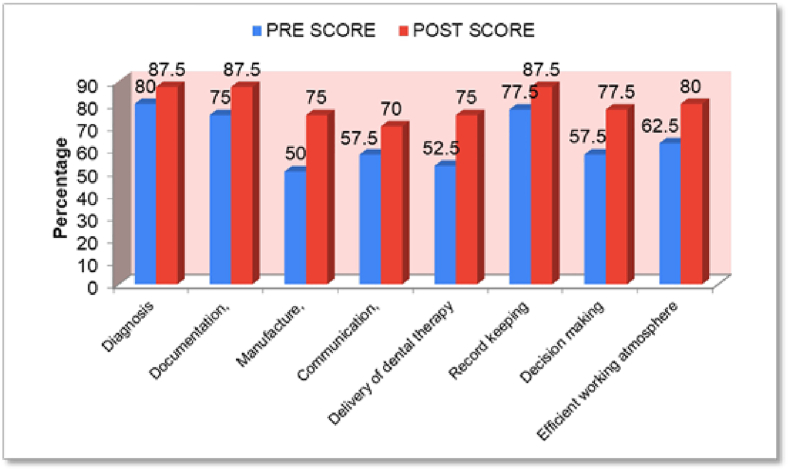
Awareness of the participants related to digitalization in various aspects of dentistry.

Graph 2 shows awareness of the participants related to digitalization in various aspects of dentistry and an increase in the awareness of participants in various aspects of digital dentistry was seen.

**Graph 3:**
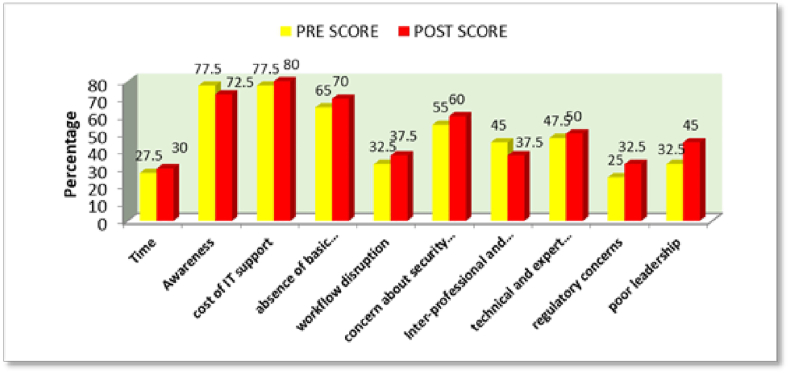
Awareness of the participants related to Recognizable barriers for Digital dentistry.

Graph 3 shows awareness of the participants related to recognizable barriers for Digital Dentistry and post-intervention, their awareness regarding recognition of barriers to digital dentistry shifted slightly.

Thematic evaluation of expectation of the participants, asked in pre-questionnaire form, regarding the Digital dentistry Module has been shown in Table 3. Themes were mainly divided into four and most of the participants agreed that they want to be aware with current advancement in the dentistry and they want to learn new procedures and the module will fulfil their expectations.

**Table 3:** Themes and verbatim responses of the participants regarding the expectation for the module.

Sr no	Themes	Verbatim
1.	Aware with current advancement/future in Dentistry	“I hope this digital dentistry will bring good future in dentistry.” “Get awareness about it so that we can easily access this in future.” “To know current advancement in dentistry.” “To know what is going in world in dentistry now a day and see future of dentistry.” “I have fair expectations with digital dentistry in the upcoming future.”
2.	Learning new things/procedures in dentistry	“I Will get to learn something new.” “To learn something new and important.”
3.	Learn various aspects of dental care	“My expectation from this course is to provide various aspects of dental care.”
4.	Improving skill	“better working environment”

Table 4 demonstrates the thematic analysis of students’ feedback asked in post-questionnaire. 12 themes were extracted from the responses and most of them responded that the module helped in learning newer technology and was very informative.

**Table 4:** Themes and verbatim responses of the participants regarding the feedback for the module.

Sr no	Themes	Verbatim
1.	learning about newer technologies	“It was good experience learning about newer technologies and their implementation in the field of dentistry.” “It's was very informative and got to see upcoming technology in dentistry” “It helped me to learn a new and interesting aspect of dentistry.” “I really gain new things and I would like to learn more regarding digital dentistry in the future” “Great experience learning about new advances and different applications of new age dentistry.”
2.	Satisfaction with the module	“Very nice”, “Good-2”, “Nice”, “Very Good-2”, “It was very helpful”, “It was very helpful and worthy session”, “Excellent”, “This was a wonderful module”, “Need more knowledge” “When I read about any big international institution related to dentistry, digital dentistry is the thing that always caught my attention. On social media also, I have seen how digital dentistry is gaining so much popularity in the developed countries but our country is still lacking somewhere in it. It feels so nice and it was also so much motivating that we were taught this wonderful course.”
3.	Informative	“This course was very useful. It helps me gain knowledge regarding digital dentistry”, “It was informative”, “Great learning and experience.” “It has given me more knowledge based on this area.” “It was very helpful. We learnt a lot from the lecture and lab visits “ “I gained lot of information through this workshop, and want to gain more such informative knowledge in future related to digital dentistry.” “Digital dentistry is a very good step towards proper and easier diagnosis and treatment for the patient and for the doctor.”
4.	Future of Dentistry	“It will be the future of dentistry” “The course was very useful in understanding the future of Dentistry” “Surely this program has provided me with a broader vision about advancements in the field and I consider it as the base of my future technically advanced clinical practice.” “I feel digital dentistry is the future of dentistry and this module certainly helped a lot in providing information and detailed knowledge about its application.”
5.	Good initiative taken	“Great initiative-2”, “It's a good initiative” “A great initiative to provide clarity on the concept of digital dentistry to UG level students.”
6.	Enhanced communication	“Digital dentistry has revolutionized the field by enhancing precision, efficiency, and patient experience. From digital impressions to CAD/CAM technology for crowns and bridges, it streamlines processes and improves outcomes. Plus, it allows for better communication between dentists and dental laboratories. However, like any technology, it requires ongoing training and investment to stay updated.”
7.	Time saving	“Digital dentistry has reduced time for technical or clinical procedures, improved quality and patient comfort.”
8.	Interactive	“Very helpful, interactive and worthy”
9.	Well organized	“Very well-organized module for embedding the basics of Digi-Den”
10.	Mandatory course	“This should be a mandatory course”
11.	Learning different aspects of dentistry	“Thanks for this great opportunity to get so many insights about digital dentistry.”
12.	Easy way Learning	“Has given us an opportunity to learn something new with easy format.”

Table 5 shows Student's performance after the session, recorded by MCQs based on the taught module showing good percentage of marks in all 3 chapters taught.

**Table-5:** Student's (40) assessment Record for the ‘Digital Dentistry Module’.

Sr no	Chapters	Total points	Mean of marks obtained	Median of marks obtained
1	Introduction of Digital dentistry	10	7.27	8
2	Diagnosis and treatment planning	10	6.66	7
3	CAD/CAM	10	7.38	8
	Total	30	7.10	8

## 4 Discussion

Innovative approaches to curriculum design and implementation are critical in meeting the needs of today's students. In the present study, an instructional Module of ‘Digital Dentistry’ was used to sensitize the undergraduate students with digital dentistry.^10^ The current module recommends 14–15 h of teaching students for making them well versed with digital dentistry. However, while introducing the module, it was felt that it is not possible to get these hours during the already existing curriculum of Bachelor of Dental Surgery, recommended by Dental Council of India. To fill that gap in duration, self-directed learning was thought to be the best option to assist the module duration along with inculcating the habit of self-directed learning amongst the students. We added self-directed learning hours which was done by the participants, in the total duration of the module.

The word SDL means-self (learner oriented), directed (facilitated and monitored), and learning (applicable to lifelong learning).^12^ Self-directed learning is one of the important pieces of the mosaic forming the knowledge base of adult learning.^13^ SDL can be used at all levels of learning, i.e., UG, postgraduates, and faculty development. Its most interesting application is at the UG level as it is an apt time to inculcate SDL skills that can continue to develop and help in learning during postgraduation and beyond as a faculty or professional.^13^ The attributes to analyse the different types of learning needs, readiness for self-directed learning, and correlation with personal learning plans shall be deliberated.^14^

Before introducing SDL module, Self-directed learning instrument^11^ was used. Total median score of SDLI was 77.325, which was a good score for the dental undergraduates. It showed student's learning motivation scored highest amongst other 4 parameters. The maximum score was 4.5 for item 3 (i.e. “I strongly hope to constantly improve and excel in my learning.“) and their planning and implementation scored lowest overall however the minimum score of was 3.29 for the item 19 in interpersonal communication (i.e. “I am able to express messages effectively in oral presentations.“).

Student's planning and implementation showed that 75 % students were able to priorities their learning (mean 4.1) and 70 % responded that they can proactively establish their learning goals (mean 3.83). The finding suggests that students require some guidance and support for planning and implementing their learning process.

Self-monitoring skills when evaluated it was found that they were aware of their strengths and weaknesses (mean 4) and were able to monitor their learning process (mean 3.83) however only 52,5 % students said that they were weak in evaluating their own learning outcome. This interprets that for evaluating learning outcomes, students require guidance.

Interpersonal communication skill evaluation showed that only 38.4 % students believed that they were able to express messages effectively in oral presentations (mean 3.29) however 65 % of students responded that they were comfortable with writing messages during communication (mean 3.8). These finding suggested that students believe to maintain good interpersonal relationship however somewhat lacking in their expression of feelings mainly during oral presentation.

The results of the present study coincide with the study done by **Siraja et al**,^15^ who assessed the SDL abilities of second-year medical undergraduates in 2024 and found that the majority of the students (61 %) demonstrated a high level of SDL ability, with a median score of 76. Though, students exhibited strong learning motivation (mean score 4.11) yet they struggled with planning and implementation (mean score 3.07). In a study done by **Bhandari et al in 2020,**^16^ the maximum score was 4.70 for *item* 3 that states, “I strongly hope to constantly improve and excel in my learning.” The minimum score of 3.74 was given to *item* 19 that states, “I am able to express messages effectively in oral presentations.” The students get motivated for learning by success and failures (mean 4.54) but lag behind in their interpersonal communication skills (mean 3.74).

After administering pre-module questionnaire, a pre designed module^16^ was applied on the volunteers. Table 2 depicts that participant showed significant improvement in their awareness and perceptions of digital dentistry. The mean digital awareness score increased from 3.33 (1.14) pre-intervention to 4.48 (0.68) post-intervention, with a mean difference of −1.15, reflecting a 34.59 % change (95 % CI: −1.49 to −0.81, Z = −4.74, p < 0.001). Agreement that digital technologies enhance communication clarity increased slightly from 3.83 (0.81) to 4.3 (0.79), with a mean difference of −0.48, a 12.42 % change (95 % CI: −0.77 to −0.18, Z = −2.92, p = 0.003). Students were Willing to adopt digital dentistry in future practice and this too improved from 4.2 (0.85) to 4.63 (0.70), with a mean difference of −0.43, a 10.12 % change (95 % CI: −0.75 to −0.1, Z = −2.44, p = 0.015).

Thematic evaluation of expectation of the participants, asked in pre-questionnaire form, regarding the Digital dentistry Module has been shown in Table 3. Themes were mainly divided in to four and most of the participants agreed that they want to be aware with current advancement in the dentistry and they want to learn new procedures and the module will fulfil their expectations. The participants responded on different manners- “Get awareness about it so that we can easily access this in future.“, “I have fair expectations with digital dentistry in the upcoming future.“, “I Will get to learn something new.” One participant responded that through the course he will learn various aspects in dentistry- “My expectation from this course is to provide various aspects of dental care.“. They all agreed that learning Digital Dentistry will; improve their skill in terms of “better working environment”.

Table 4 demonstrate the thematic analysis of students' feedback of the taught module asked in post-questionnaire. 12 themes were extracted from the responses and most of them responded that the module helped in learning newer technology and was very informative. Few of the verbatims recorded were- “I really gain new things and I would like to learn more regarding digital dentistry in the future”, “Great experience learning about new advances and different applications of new age dentistry.” “I gained lot of information through this workshop, and want to gain more such informative knowledge in future related to digital dentistry.” “Digital dentistry is a very good step towards proper and easier diagnosis and treatment for the patient and for the doctor.” Many of the students gave the feedback in terms of satisfaction with the module saying “Very nice”, “Good”, “Nice”, “Very Good”, “It was very helpful”, “It was very helpful and worthy session”. One of the students also showed his prior interest in the module saying- “When I read about any big international institution related to dentistry, digital dentistry is the thing that always caught my attention. On social media also, I have seen how digital dentistry is gaining so much popularity in the developed countries but our country is still lacking somewhere in it. It feels so nice and it was also so much motivating that we were taught this wonderful course.” Few agreed that digitalization is the future of dentistry with the received verbatims like- “Surely this program has provided me with a broader vision about advancements in the field and I consider it as the base of my future technically advanced clinical practice.” and “I feel digital dentistry is the future of dentistry and this module certainly helped a lot in providing information and detailed knowledge about its application.” Few praised the module conduction and said - “It's a good initiative”, “A great initiative to provide clarity on the concept of digital dentistry to UG level students.” One of the students said that digitalization helps in saving time- “Digital dentistry has reduced time for technical or clinical procedures, improved quality and patient comfort.” Few of the participants responded as it was a well-organized course and it should mandatory for undergraduate students. Few responded that they learnt different aspects of dentistry in an easy way.

In the present module, student's performance after the session, recorded a good percentage of marks in all 3 chapters taught. Mean of total scores obtained was found to be around 70 %, which clearly show the improvement in the knowledge of the students regarding Digital dentistry. **Rajalakshmi and Ganapathy**^9^ have also used similar module while assessing the effect of the SDL module on “National Health Programme” in Community Medicine. The students learned new terminologies in NHP and responded that the module stressed difficult topics in the curriculum. The students felt that SDL module was the simple and easily understandable module, and peer-discussion during activities was the facilitating factors.

Digital technology has the potential to transform dental education, and it may assist future dentists in preparing for their everyday practices.^17^ In the present study, mixed-methods design for implementation of module, a validated SDL instrument, and integration of a digital dentistry module into an existing curriculum were managed to be taught to the student within a specific time limit. Innovative approaches to curriculum design, like including self-directed learning in the present case, helped the module to achieve its objectives while also providing a more personalized and effective learning experience for students. Various Multimedia and experiential learning approach like using google forms for feedback and assessments, video demonstration, sharing of available literature along with demonstration of available digital equipment in the institution have helped the student to improve better understanding of the subject are the core of the study. As a beginner course, the project could fulfil its objectives and outcome achieved was quite satisfactory. However, due to limited resources, students could not experience personal practical learning, though demonstration of few of the techniques was done in the lab. As the study was done in a single institution, the result obtained cannot be generalised to the population and further multicentric study of this kind is required for external validation. The conduct of the study provides practical relevance to real-world academic settings as this is the era of digitalization and there is a need of these kind of studies to improve the quality of education.

## 5 Conclusion

Within the limitations of the present study, following conclusions can be drawn-

1. Student's self-directed learning abilities, as evaluated by using SDLI score with median value of 77.325 showed a good score.
2. After the module completion, Participants showed significant improvement in their awareness and perceptions of digital dentistry. The total score significantly improved post-intervention, with the mean score increasing from 28.83 (5.43) pre-intervention to 35.35 (4.36) post-intervention.
3. Outcome assessment showed mean of total scores around 70 %, representing good performance of the students in the ‘Digital Dentistry’ module.

As a beginner course in Dental undergraduate curriculum, the present study could fulfil its objectives and outcome achieved was quite satisfactory. However, as the study was limited to a single institution involving limited number of participants, further multicentric study is required for its implementation and validation on wide population.

## Declaration of generative AI and AI-assisted technologies in the writing process

NA.

## Source of funding

This research did not receive any specific grant from funding agencies in the public, commercial, or not-for-profit sectors.

## Declaration of competing interest

The authors declare that they have no known competing financial interests or personal relationships that could have appeared to influence the work reported in this paper.
