# Supplementary material for: Assessment of students’ perspective on introduction of “digital dentistry” as self- directed learning module in undergraduate prosthodontics curriculum: A mixed-method study
Source: J Oral Biol Craniofac Res. 2025 Jun 22;15(5):925–31. doi: 10.1016/j.jobcr.2025.06.010 (PMC12226062; doi:10.1016/j.jobcr.2025.06.010)
Supplement: Multimedia component 1 [file mmc1.docx]

**Resource material shared in the study-**

- https://www.clinicalkey.com/#!/content/book/3s2.0B9780323809757000049?scrollTo=%23hl0000498
- https://www.clinicalkey.com/#!/content/journal/1-s2.0-S2212426820300427
- https://www.ncbi.nlm.nih.gov/pmc/articles/PMC5093293/#:~:text=The%20introduction%20of%20a%20whole,for%20planning%20implant%20surgery)%2C%20together
- https://youtu.be/Ev54zSIaIQs?si=-UbIGkprAN50zfbd
- https://youtu.be/FZeAiGV9xIU?si=A6zYxwOwQ9zDod_f
- https://youtu.be/af1t9kFgVp0?si=RYQ8ZMwZ4OxwL3QQ
